# Mycotoxins at the Start of the Food Chain in Costa Rica: Analysis of Six *Fusarium* Toxins and Ochratoxin A between 2013 and 2017 in Animal Feed and Aflatoxin M_1_ in Dairy Products

**DOI:** 10.3390/toxins11060312

**Published:** 2019-05-31

**Authors:** Andrea Molina, Guadalupe Chavarría, Margarita Alfaro-Cascante, Astrid Leiva, Fabio Granados-Chinchilla

**Affiliations:** 1 Centro de Investigación en Nutrición Animal (CINA), Universidad de Costa Rica, Ciudad Universitaria Rodrigo, San José 11501-2060, Costa Rica; andrea.molina@ucr.ac.cr (A.M.); molinaucr@gmail.com (G.C.); alfarocascante@gmail.com (M.A.-C.); astrid.leiva@ucr.ac.cr (A.L.); 2 Escuela de Zootecnia, Universidad de Costa Rica, Ciudad Universitaria Rodrigo, San José 11501-2060, Costa Rica

**Keywords:** *Fusarium* mycotoxins co-contamination, ochratoxin A, feed prevalence and safety, HPLC analysis

## Abstract

Mycotoxins are secondary metabolites, produced by fungi of genera *Aspergillus*, *Penicillium* and *Fusarium* (among others), which produce adverse health effects on humans and animals (carcinogenic, teratogenic and immunosuppressive). In addition, mycotoxins negatively affect the productive parameters of livestock (e.g., weight, food consumption, and food conversion). Epidemiological studies are considered necessary to assist stakeholders with the process of decision-making regarding the control of mycotoxins in processing environments. This study addressed the prevalence in feed ingredients and compound feed of eight different types of toxins, including metabolites produced by *Fusarium* spp. (Deoxynivalenol/3-acetyldeoxynivalenol, T-2/HT-2 toxins, zearalenone and fumonisins) and two additional toxins (i.e., ochratoxin A (OTA) and aflatoxin M_1_ (AFM_1_)) from different fungal species, for over a period of five years. On the subject of *Fusarium* toxins, higher prevalences were observed for fumonisins (*n* = 80/113, 70.8%) and DON (*n* = 212/363, 58.4%), whereas, for OTA, a prevalence of 40.56% was found (*n* = 146/360). In the case of raw material, mycotoxin contamination exceeding recommended values were observed in cornmeal for HT-2 toxin (*n* = 3/24, 12.5%), T-2 toxin (*n* = 3/61, 4.9%), and ZEA (*n* = 2/45, 4.4%). In contrast, many compound feed samples exceeded recommended values; in dairy cattle feed toxins such as DON (*n* = 5/147, 3.4%), ZEA (*n* = 6/150, 4.0%), T-2 toxin (*n* = 10/171, 5.9%), and HT-2 toxin (*n* = 13/132, 9.8%) were observed in high amounts. OTA was the most common compound accompanying *Fusarium* toxins (i.e., 16.67% of co-occurrence with ZEA). This study also provided epidemiological data for AFM_1_ in liquid milk. The outcomes unveiled a high prevalence of contamination (i.e., 29.6–71.1%) and several samples exceeding the regulatory threshold. Statistical analysis exposed no significant climate effect connected to the prevalence of diverse types of mycotoxins.

## 1. Introduction

Mycotoxins are toxic fungal metabolites that can be found in feed ingredients and compound feeds [1,2]. Due to their compositions, they are detrimental to animal and human health [3,4,5,6,7,8]. Currently, more than 400 different types of mycotoxins have been identified [9]. However, *Fusarium* toxins are among the most commonly monitored as they are acknowledged to present serious health concerns [7,10]. Under certain conditions, some fungi can produce several toxins simultaneously [11,12,13]. 

In feed production, ca. 60% of the formulation consists of cornmeal, soybean meal, and their derivates [14,15]. In Costa Rica, cereal production represents 38% of the agricultural sector imports [16], where its main suppliers are the United States and Brazil with 84% and 15% contribution, respectively [17]. In this regard, corn imports have increased from 738,539.97 to 781,903.54 metric tons from 2015 to 2017 [18]. On the other hand, soybean imports have risen to 309,897.97 metric tons per year, even though 83% of the soybean meal used as a feedstuff comes from national production [18]. Furthermore, only 38% of the products destined for animal consumption are from national origin, representing a total feed production of 1,238,243 metric tons in 2017. Approximately 45%, 27%, 20%, and 4% of this production is intended to be destined to poultry, higher ruminants, swine, and pets (i.e., cats and dogs), respectively [18]. That is, import and export of animal feed and feed ingredients play an essential part in the co-occurrence of various types of mycotoxins in the finished feed [19,20]. Hence, co-occurrence could be a far more certain and prevalent issue in real mycotoxin feed analysis [11,12,20,21,22,23]. 

Mycotoxin metabolites retain toxicity and thus must be surveilled [24,25]. Mycotoxins and their metabolites have several implications for animal and human health. Some are identified/classified as teratogenic, genotoxic, carcinogenic, and immunotoxic. The ingestion of contaminated feed affects animal health and may reduce productivity in animals, generating economic losses [26]. Some mycotoxins ingested and metabolized by productive animals could be accumulated in different organs and tissues reaching the food chain through meat, milk, or eggs [24,27,28]. In Costa Rica, during 2018, consumption of these commodities was estimated in 58.7 kg (i.e., 14.3, 15.4, and 29 kg year^−1^ for cattle, pork, and chicken, respectively), 215 L, and 218 units per capita, individually [18]. 

In this regard, epidemiological information tends to be more comprehensive when exploring data from several toxins simultaneously [29]. Accurate mycotoxin data about their presence in feeds are paramount for stakeholders’ decision-making process towards the risk management in their manipulation [30]. Numerous reports have explicitly documented the incidence of mycotoxins in feeds, especially in Europe [11,31,32], USA [33], Asia [31], and China [34]. Nowadays, there are insufficient reports oriented to describe the incidence of mycotoxins in feed in Costa Rica. The emphasis has been made towards the investigation of aflatoxins [35,36]. 

Herein, the prevalent data from feed and feed ingredient samples of eight different toxins, mainly produced by *Fusarium* spp. (deoxynivalenol/3-acetyldexoynivalenol (DON/3-ADON), T-2/HT-2 toxins, zearalenone (ZEA) and fumonisins (FB_1_ and FB_2_)), but also ochratoxin A (OTA), during five years are provided. Finally, in the same period, we analyzed the behavior of AFM_1_ in liquid milk. 

## 2. Results

### 2.1. Fusarium Toxins Present in Animal Feed

The highest prevalence of *Fusarium* toxins during the analyzed period (2012–2017) was observed for fumonisin and DON in 70.8% (*n* = 80/113) and 58.4% (*n* = 212/363) of the cases, respectively. For FB_1_ + FB_2_ the prevalence ranged from 27.8% (*n* = 5/18) in 2013 to 85.2% (*n* = 23/27) in 2014, with a maximum concentration of 53,580 µg kg^−1^ observed in 2015. The prevalence for DON ranged from 42.0% (*n* = 40/94) in 2016 to 79.3% (*n* = 69/87) in 2014, with a maximum concentration of 151,060 µg kg^−1^ presented in 2013 (Table 1). Lower prevalences of 21.2% (*n* = 45/212) and 36.1% (*n* = 97/269) with a maximum mycotoxin level of 16,100 µg kg^−1^ (in 2015) and 12,500 µg kg^−1^ (in 2014) were observed for 3-acetyldeoxynivalenol and HT-2, respectively (Table 1). Concentration-wise and among periods, ZEA and T-2 toxin increased meaningfully in 2017 and 2013, respectively. For HT-2, OTA, DON, 3-ADON, FB_1_, FB_2_, and FB_1_ + FB_2_, no differences were observed.

### 2.2. Mycotoxin Prevalence in Feed Ingredients

In the matter of feed ingredients, cornmeal exceeded guideline values for HT-2 toxin (*n* = 3/24, 12.5%), T-2 toxin (*n* = 3/61, 4.9%), and ZEA (*n* = 2/45, 4.4%) (Table 2). In a soybean meal, merely HT-2 toxin (*n* = 1/6, 16.7%) was detected in this situation, and just one sample of wheat had an excessive amount of DON (*n* = 1/8, 12.5%) (Table 2). With reference to other raw materials, of less inclusion, such as rice byproducts, palm oil byproducts, of the citrus industry, as well as forages, silages, and hays (treated as a whole group), there are no regulatory guidelines to establish an acceptance parameter. However, it is interesting to notice that, in the groups described above, they share as a common feature a high prevalence of DON (i.e., 66.7%) (Table 2). 

### 2.3. Mycotoxin Prevalence in Compound Feed

Among compound feeds, beef cattle feed presented only a few samples above the guideline level (specifically, T-2 and HT-2 toxin, *n* = 2/63, 3.2%). Dairy cattle feed presented the highest number of samples that surpassed the recommended levels of mycotoxins (*n* = 34/105, 32.4%), specifically DON (*n* = 5/147, 3.4%), ZEA (*n* = 6/150, 4.0%), T-2 toxin (*n* = 10/171, 5.8%) and HT-2 (*n* = 13/132, 9.8%) (Table 3). Poultry feed presented only 10 samples exceeding the guidelines, for DON (*n* = 2/14, 14.3%), FB_1_ (*n* = 1/7, 14.3%), HT-2 toxin (*n* = 1/15, 6.7%), and OTA (*n* = 1/9, 11.1%). Cat and dog food also showed values above legal thresholds for fumonisins (*n* = 6/13, 46.1%), with a maximum of 18,910 µg kg^−1^ (Table 3). The second highest prevalence was observed connected with swine feed (*n* = 14/71, 19.7%) with the mycotoxins ZEA (*n* = 2/18, 11.2%), FB_1_ (*n* = 2/9, 22.2%), and DON (*n* = 6/17, 35.3%) infringing the respective recommended guidelines (Table 2). Fish feed also exceeded thresholds for DON (*n* = 2/16, 12.5%). Finally, in horse feed, Fumonisin B_2_ was found (*n* = 1/26, 3.8%) (Table 3).

### 2.4. Geographical Distribution and Climate Influence for Fusarium Toxins Present in Animal Feed

**Figure 1:**
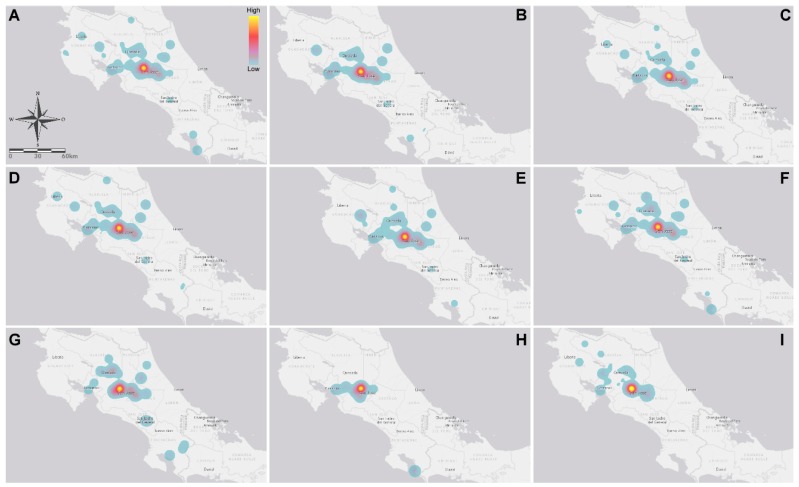
Heat map representing the geographical origin of samples and the mycotoxin concentration: (**A**) DON; (**B**) 3-ADON; (**C**) T-2 toxin; (**D**) HT-2 toxin; (**E**) ZEA; (**F**) FB_1_; (**G**) FB_2_; (**H**) OTA; and (**I**) AFM_1_.

Geographical and national toxin hotspot distribution was similar for those toxins produced by *Fusarium* species (Figure 1A–G). A completely different profile was observed when studying OTA and AFM_1_. Interestingly, only 3-ADON and HT-2 toxins prevailed during the rainy season. For other toxins, there were no differences in the levels of contamination between the dry season and the rainy season (Table 4). As expected, the co-occurrence of two different toxins was the most common situation (i.e., *n* = 141/279, 50.5%) (Table 5). Therefore, as the number of simultaneous toxins increased, co-occurrence was less likely to be found (Table 5). In the case of the parent compound–metabolite comparison, the most common combination was the pair T-2/HT-2 toxin with (*n* = 66/155) 42.6% of prevalence, followed by FB_1_/FB_2_ (*n* = 23/137, 16.8%) and DON/3-ADON (*n* = 18/177, 10.2%) (Table 5).

### 2.5. OTA Prevalence in Animal Feeds

**Figure 2:**
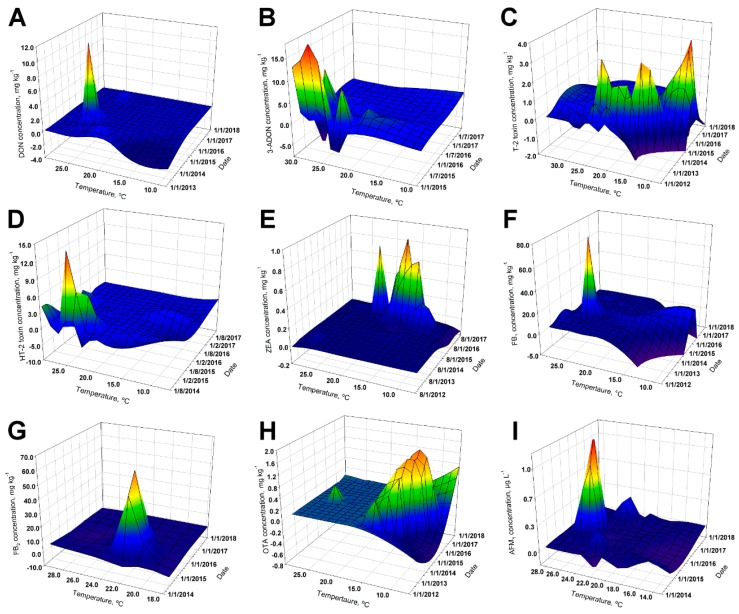
3D mesh graphs representing the relationship among mycotoxin concentration, mean temperature, and sample date: (**A**) DON; (**B**) 3-ADON; (**C**) T-2 toxin; (**D**) HT-2 toxin; (**E**) ZEA; (**F**) FB_1_; (**G**) FB_2_; (**H**) OTA; and (**I**) AFM_1_.

Referring to OTA, the total prevalence from 2012 to 2017 was 40.6% (*n* = 146/360), ranging from 16.3% (*n* = 8/49) in 2013 to 76.6% (*n* = 49/64) in 2015. The maximum OTA reported level was 1810 µg kg^−1^, in 2016 (Table 1). Only one sample exceeded the maximal advisory level for ochratoxin; this sample corresponded to poultry feed where the recommended concentration is 100 µg kg^−1^. The overall OTA prevalence in non-traditional ingredients, poultry, and fish feed was of 56.3%, 44.4%, and 66.7%, respectively (Table 2 and Table 3). Furthermore, in May and September, the highest global concentrations of OTA were presented, corresponding to the rainy season releasing an evident difference compared with the findings of the dry season (Table 4). As the presence of OTA involves other toxin-producing fungi (other than *Fusarium*), co-occurrence with other metabolites is a possibility. The most prevalent *Fusarium* toxins present in feed (different from OTA), in decreasing order of incidence, were ZEA, DON + FB_1_, FB_1_, and T-2 toxin with (*n* = 17/102) 16.7%, (*n* = 14/102) 13.7%, and (*n* = 12/102) 11.8% of incidence, respectively (Table 5). As expected, OTA incidence had a completely different geographical/spatial (Figure 1H) and thermo/temporal (Figure 2H) distribution, when compared with the other toxins.

### 2.6. Aflatoxin M_1_ in Liquid Milk

Water buffalo milk and butter samples were also analyzed for the presence of Aflatoxin M_1_. Water buffalo (*Bubalus bubalis*) milk samples (*n* = 2) were reported below the limit of quantification (i.e., 0.014 µg kg^−1^) and butter (*n* = 3) ranged from 0.021 to 0.024 µg kg^−1^. Even though 2016 was the year with the lowest number of analyzed samples, it was also the year when fewer samples surpassed the 0.05 µg kg^−1^ threshold (Table 6). An increase in AFM_1_ prevalence with 71.1% and 63.2%, respectively (Table 6), was observed during 2014 and 2017. Excluding three samples from 2015, there were no other samples surpassing the US FDA threshold of 0.5 µg kg^−1^, thus representing a very small overall percentage for the four years of the study (i.e., *n* = 3/175, 1.7%). It was studied/monitored that, consistently, higher concentrations of AFM_1_ were obtained during March, August, and September (Table 6 and Figure 2I).

## 3. Discussion

### 3.1. Mycotoxin Prevalence between 2013 and 2017 in Animal Feed

Most of the studied toxins (except for 3-ADON, FB_1_, and HT-2) had prevalences higher than 40% during the five years. The average concentrations found in the different toxins in animal feed did not vary between one year and another, except for ZEA and T-2. The drastic increase of ZEA concentrations during 2017 was observed in corn meal and sorghum silo. There is a prior documented avidity of *Fusarium* spp. to produce ZEA when using moderately alkaline cereals (e.g., maize) as substrates [43]. A general drop in annual temperature may have provoked this upsurge in ZEA contamination. For example, *Fusarium graminearum* has demonstrated that conditions of pH 9 and incubation temperature of 15.05 °C are required to favor ZEA production [44]. Interestingly, the most toxicologically relevant levels for ZEA were encountered at relatively low temperatures (i.e., near 15 °C). Despite a relatively high prevalence for mycotoxins (i.e., between 46% and 99%, except for FB_1_ + FB_2_ and DON), the positive samples possessed comparatively low concentrations (Table 1) based on guidance values for mycotoxins in animal feeds within the European Union (see Appendix A Table A1 and Table A2) [37,38]. This relatively low toxicological burden could be associated with the control of mycotoxin in animal feed and raw materials that were established in the country since 2007. This control policy covers the majority of the toxins analyzed in this study added to the control of imported raw materials, before its distribution. In coherence to what has been stated, since 2013, proficient manufacturing practices have been evaluated and audited by regulation in animal feed plants. These proficient practices involve the management of raw materials and storage measures, among others, contributing to the reduction of mycotoxin contamination [45]. 

However, some of the samples were observed with concentrations above the established guidelines with potentially adverse effects on animal health and productivity. It is worth of mentioning the fact that human health could be affected through the consumption of foods of animal origin contaminated with mycotoxins or their metabolites [24,27,28]. 

### 3.2. Mycotoxin Prevalence in Compound Feed and Feed Ingredients

#### 3.2.1. Prevalence in Feed Ingredients

Vegetable ingredients may represent from 80% to 100% of the feed (e.g., in ruminants, animal origin ingredients are prohibited) [14,46,47]. For these vegetable-based formulations, corn and soybean meal may represent up to 60% of the input [14,15]. Costa Rican soybean meal and corn, as well as other relevant ingredients, are imported [18]. Quality grain assessment is a degree-based classification. Usually, grade 2 or 3 corn is purchased for feed production [18]. At least 97.9% of the samples contain around 3% of cracked material, and 36.2% of the samples exhibited higher moisture content (i.e., 17%); both factors promote the proliferation of fungi [48]. Toxin-wise, AFB_1,_ and DON were assayed and are regulated according to FDA criteria. Only 1.9% samples exceeded levels for AFB_1_ but none for DON [49]. The data reveal coherence with the obtained results (Table 2). Notwithstanding, a high prevalence for DON was detected and reported by other researchers both for corn and wheat [49]. Conversely, a relatively lower incidence was found in OTA, different from what was conveyed elsewhere [50]. 

#### 3.2.2. Prevalence in Cattle Feeds

In both dairy and meat cattle, forage, hay, and silage input must not be underplayed, especially in countries where extensive feeding systems based on grazing cattle predominate. Considering Costa Rica a particular case, 85% and 95.9% of the dairy and beef cattle are based on grazing farming, respectively [51]. Relatively favorable toxin profiles were still found in the tested samples. Thereby, surveillance efforts have been focused on compound feed. Generally speaking, ruminants are relatively less sensitive toward the effects of mycotoxins as rumen bacteria play a detoxification role [35,38]. For example, for DON (prevalence of 70.0% and 55.1% in beef cattle feed and dairy cattle feed, respectively), Charmley and collaborators determined that concentrations of 6000 μg kg^−1^ neither affect feed intake nor are biotransferred to the milk [36,52].

#### 3.2.3. Prevalence in Compound Feed destined for Poultry and Swine

Mycotoxin effects over monogastric animals are varied, depending on the species and physiological and productive stage [53]. For example, in pigs, fumonisin feed contamination is related to pulmonary, hepatic and cardiovascular lesions [54] while DON has been associated with a reduction of productive parameters and feed efficiency [54]. Besides, pigs are especially sensitive to ZEA, as it is directly related to reproductive disorders and low fertility rates [55]. Mycotoxin findings in poultry feed are also worrisome as birds are noticeably susceptible to molecules such as DON. For example, in broilers, trichothecene exposure (e.g., DON), through feed, increases mortality, reduces immune function, and impairs weight gain [56]. 

#### 3.2.4. Prevalence in Pet Food

Mycotoxins in pet foods have already been reported by other countries, including industrialized ones (e.g., Portugal, USA, England, and Brazil) [57]. Mainly, *Fusarium* and *Penicillium* toxins have been described [51]. An elevated prevalence was described for DON and FB_1_ (50.0% and 93.3%, respectively) [58]. Mycotoxicosis in pets is associated with chronic disease, liver and kidney damage, and cancer [58]. Finding mycotoxins in thermally treated foods is not uncommon as mycotoxins molecules can withstand relatively elevated temperature; low toxin reduction will occur during extrusion. Fungi colonization of pet extruded food is expected to be low as it possesses relatively low values of moisture and water activity [58,59]. Mycotoxin in pet foods may represent an additional burden to humans due to the pet closeness with their owners. 

#### 3.2.5. Prevalence in Fish Feed

Presence of mycotoxins in fish feed is another proof of an industry which has progressively substituted animal protein sources for vegetable ones [60,61]. In this regard, DON, OTA, and ZEA have been said to be responsible for weight loss, exacerbated feed conversion, and increased susceptibility to infection and disease in fish [61,62]. In line with the data reported herein, a recent report revealed that commercial fish feed samples were frequently contaminated with DON (i.e., over 80% of the samples) with mean concentrations of 289 μg kg^−1^ [49]. Levels as low as 4.5 mg DON kg^−1^ feed have already confirmed adverse effects in productive parameters and increased mortality in some fish. even in a relatively short period [62]. 

### 3.3. Geographical Distribution and Climate Influence for Fusarium Toxins Present in Animal Feed

A different spatial distribution profile was observed for AFM_1_ and OTA, which are not produced by *Fusarium* species. *Fusarium* species have the potential of simultaneously producing the remainder of the toxins assayed [63,64]. OTA is a toxin produced by several fungal species including *Aspergillus ochraceus*, *A. carbonarius*, *A. niger* and *Penicillium verrucosum* [65]. On the other hand, AFM_1_ is not only produced by *Aspergillus* species but it is also a product of metabolism [66]. Our data not only demonstrate that most sampling weight is centered on the Costa Rican Central Valley plateau, but the largest concentrations also occur therein (geographical zones with a high average relative humidity of 82%). The data also demonstrate that the intricate climate in tropical countries (such as Costa Rica) predicts the behavior of mycotoxin contamination as more challenging.

### 3.4. Aflatoxin M_1_ in Liquid Milk

Milk is not only a staple commodity by itself, but it can accompany other potentially contaminated products (e.g., coffee, tea, or chocolate). Additionally, although AFM_1_ is the most studied toxin in milk, other toxins have been described as well [67]. Other dairy products are derived from this raw material (e.g., cheese). Although processing is involved, these other dairy products can carry by themselves aflatoxin metabolites as well (see, for example, [68]). During 2017 alone, milk consumption was calculated to be 212 kg per capita [18]. Assuming the worst-case scenario (a sample with the highest concentration of 0.989 µg kg^−1^), a Costa Rican citizen could be exposed up to 210 µg AFM_1_ per year. Similarly, a Jersey calf weighing 25–30 kg at birth would be fed with 10% of its live weight with contaminated milk (from 2.5 to 3 kg of milk per day) [69]. Reiteratively, this means a daily exposure of 2.5–3 µg AFM_1_ per day. Milk weaning can occur at ten weeks old [70]. Milk consumption level exposure is estimated to be 0.023 ng AFM_1_ per kg body weight per day when a maximum level of 0.5 μg kg^−1^ is used.

Much higher average concentrations of AFM_1_ have been documented in other Latin-American countries [71]. Interestingly, AFB_1_ (the parent compound of AFM_1_) has been reported to be present in milk samples [71]). Besides the toxic burden that AFB_1_ and AFM_1_ have in the liver, recent evidence suggests that kidney toxicity is a certainty [66]. On the other hand, considerably low (i.e., 0.037 µg kg^−1^) AFM_1_ levels in milk have been recently reported, although prevalence rates are also relatively high (i.e., 38.8%), [71]. Other Latin-American countries have reported similar percentages [72,73,74,75], and recent prevalence studies have been published in industrialized countries [76,77,78,79]. Epidemiological studies [1] and risk assessment [80,81,82] are paramount to reduce mycotoxin exposure to both humans and animals.

Aflatoxin-contaminated feed must also be monitored to avoid feeding dairy cows with contaminated batches [83]. For instance, the association among most aflatoxin-contaminated feed ingredients and prevalence has been detailed [36,73]. Although the samples reported herein come from a highly industrialized sector, similar prevalence has been reported in fresh milk from small farms [84]. Consistent with our results, the seasonal distribution does not seem to affect AFM_1_ prevalence [71], probably because Costa Rica has a tropical climate. In general, Costa Rica has relatively high temperatures (19–30°C), humidity (60–91%) and abundant rainfall (1400–4500 mm per year) during a great part of the year (i.e., two distinct seasons), in opposition to an Iranian study exhibited a lower prevalence of AFM_1_ in bovine milk during spring [85]. Seasonal variations (i.e., during rainy season) were also described for milk from other species (i.e., sheep, goat, and camel) [81]. Other researchers have not documented a clear tendency regarding AFM_1_ occurrence during seasons [73]. It has been suggested, however, that climate change can bear an impact on human exposure to aflatoxins and health [85]. Finally, the burden of AFM_1_ exposure for a human can be twice as much as breast milk contamination, as has also been well documented [86]. Although some methods for reducing AFM_1_ contamination are available [87], pre- and post-harvest strategies are still the most effective strategies [88]. 

## 4. Conclusions

Toxicologically relevant concentrations were found during the five-year survey as some sample concentrations exceeded the regulatory guidelines. Fumonisin and deoxynivalenol feed contamination is worrisome since these toxins have the capacity of being found in significant levels in these matrices, and, in our case, higher levels of toxins are found in the Central Valley of the country. Therefore, surveillance programs should be expanded to the outermost productive regions of the country to suppress sampling bias, if existing any. Thermopluvial conditions do not seem to have a considerable effect on toxin levels, although some metabolites actually seem to behave concurrently. *Fusarium* metabolites must be stridently monitored as it is clear that contamination in feed and feed ingredients is unfortunately common; this is especially true for fumonisins and T-2. Feed manufacturers, farmers (both in the field and storage facilities) and pet owners alike should be educated as to the proper conditions for food storage to avoid mycotoxin-producing fungal colonization. Toxin metabolite analysis and co-occurrence are paramount for complete surveillance of toxin feeds, and efficiently execute systems for the control and reduction of mycotoxins, as well as their metabolites in feeds. In addition, a strict control of AFM_1_ in milk is necessary, because the prevalence of AFM_1_ in milk is considerable and several samples exceeded the regulatory thresholds. It must be remembered that milk is the raw material for a wide variety of dairy products (butter, cheese, and yogurt, among others), therefore, the exposure of the population to this mycotoxin is increased. 

## 5. Materials and Methods

### 5.1. Reagents

An analytical standard with certified concentrations, dissolved in acetonitrile, for DON, 3-ADON, T-2 (TSL-314), HT-2 (TSL-333), ZEA (TSL-401), FB_1_, FB_2_ (TSL-202), and OTA (TSL-504) was purchased from Trilogy^®^ Analytical Laboratory Inc (Washington, MO, USA). All standards have an initial concentration of 100 mg L^−1^, except for FB_2_ that was at 30 mg L^−1^. Additionally, a naturally contaminated reference material (TRMT100, cornmeal) was used as a quality control sample (TS-108, Washington, MO, USA). Acetonitrile (ACN) and methanol (MeOH), both chromatographic grade, were purchased from J.T. Baker (Avantor Materials, Center Valley, PA, USA). Ultrapure water (type I, 0.055 µS cm^−1^ at 25°C, 5 µg L^−1^ TOC) was obtained using an A10 Milli-Q Advantage system and an Elix 35 system (Merck KGaA, Darmstadt, Germany).

### 5.2. Sampling

A total of *n* = 487 different feedstuffs of ca. 5 kg were collected during 2013–2017 by government inspectors from *n* = 107 Costa Rican feed manufacturers, as part of a countrywide surveillance program. Sample collection was composed of compound feed and feed ingredients, as follows: dairy cattle feed 28.9% (*n* = 141), cornmeal 9.9% (*n* = 48), citrus pulp 5.5% (*n* = 27), cattle feed 5.5% (*n* = 27), pig feed 5.3% (*n* = 26), calf feed 4.3% (*n* = 21), palm kernel meal 4.1% (*n* = 20), fish feed (Tilapia) 3.7% (*n* = 18), poultry feed 3.5% (*n* = 17), distillers dried grains 3.5% (*n* = 17), hay 3.3% (*n* = 16), dog food 3.3% (*n* = 16), wheat middlings 2.9% (*n* = 14), soybean meal 2.7% (*n* = 13), layer hen feed 2.0% (*n* = 10), horse feed 1.8% (*n* = 9), forage 1.8% (*n* = 7), pineapple byproducts 1.2% (*n* = 6), cassava meal 1.2% (*n* = 6), sorghum meal 0.6% (*n* = 3), rodent feed 0.6% (*n* = 3), ground roasted coffee 0.6% (*n* = 3), banana peel 0.6% (*n* = 3), rice bran 0.4% (*n* = 2), chamomile flowers 0.4% (*n* = 2), soybean hulls 0.2% (*n* = 1), shrimp feed 0.2% (*n* = 1), rice meal 0.2% (*n* = 1), rabbit feed 0.2% (*n* = 1), hydrolyzed feather meal 0.2% (*n* = 1), fish feed (snapper, *n* = 1), fish feed (salmon and trout, *n* = 1), corn silage (*n* = 1), and corn gluten (*n* = 1). Selection of feed and feed ingredients to be tested, number of samples, sampling sites, and specific toxins to assay (per matrix) were chosen by feed control officials. The selection considered the most common feedstuffs used in Costa Rica, import and export regulations, contamination risk factors, the productivity of the feed industry, and the risk for human and animal health associated with each feed or feed ingredient. Sampling was performed following the Association of American Feed Control Officials (AAFCO) recommendations for mycotoxin test object collection [89], and samples were taken from silos and storage reservoirs from feed manufacturing plants. All samples were quartered and sieved (1 mm particle size) [89]. Additionally, *n* = 180 dairy samples (mostly liquid bovine milk) from *n* = 13 different Costa Rican dairy farms were assayed; 50 mL subsamples were processed from 500 mL samples. 

### 5.3. Reference Methods for Toxin Determination

Mycotoxins were assayed using the following methods: DON/3-ADON [90], T-2 and HT-2 toxins [91], ZEA AOAC 976.22, fumonisins AOAC 995.15, and OTA AOAC 991.44. AFM_1_ was assayed according to the methods in [36,92] for milk and butter, respectively. 

### 5.4. Chromatographic System and Conditions

All analytes were assayed using HPLC. Equipment consisted of an Agilent 1260 Infinity series HPLC with a quaternary pump (G1311B), a column compartment (G1316A), a variable wavelength and fluorescence detector (G1314B and G1321B) and an autosampler system (G1329A) (Agilent Technologies, Santa Clara, CA, USA). Peak separation was accomplished using a 5 mm Agilent Zorbax Eclipse C_18_ column (3.0 × 150 mm, 5 µm) except for T-2/HT-2 toxin analyses for which a Luna^®^ Phenyl-Hexyl column (4.6 × 150 mm, 5 µm) was used (Phenomenex, Torrance, CA, USA). All analytes, except AFM_1_, were extracted using Immunoaffinity columns (R-biopharm Rhöne Ltd, Darmstadt, Germany). 

#### 5.4.1. DON/3-ADON

DONPREP^®^ (R-biopharm) columns were used for sample extraction. Briefly, 200 mL of purified H_2_O was added to 25 g of test portion. The mixture was dispersed using an Ultra-Turrax^®^ (T25, IKA Works GmbH & Co, Staufen, Germany) at 8000 rpm. The supernatant was filtered by gravity over an ashless filter paper (Grade 541, Whatman^®^, GE Healthcare Life Sciences, Marlborough, MA, USA). Subsequently, an exact 2 mL aliquot from the supernatant was transferred to the IAC column and passed at 1 mL min^−1^ using an SPE 12 port vacuum manifold (57044, Visiprep™, Supelco Inc., Bellefonte, PA, USA) at 15 mm Hg vacuum. After a washing step using 2× 10 mL water, the columns were left to dry and then four MeOH fractions of 500 µL were passed through the IAC. The total volume recovered was concentrated to dryness under vacuum at 60°C. The sample was reconstituted with MeOH to 300 µL and transferred to an analytical HPLC conical vial insert (5182-0549, Agilent Technologies, Santa Clara, CA, USA) before injection into the chromatograph.

Gradient mode starting at 80:20 H_2_O, Solvent A/CH_3_OH, Solvent B as per chromatographic conditions. The rest of the program was as follows: at 0.5 min 80% A, at 5.50 min 90% A, at 10 min 90% A, at 11 min 80% A, and at 15 min 80% A. DON and 3-ADON absorption at 220 nm was exploited for detection purposes. Linear calibration curves ranging from 1.25 to 10.00 µg mL^−1^ were prepared during quantification. The limit of quantification for DON/3-ADON was 10.00 and 40.00 μg kg^−1^.

#### 5.4.2. T-2 and HT-2 Toxin

The extraction was similarly performed as detailed for DON/3ADON using an EASI-EXTRACT^®^ T-2 and HT-2 IAC (R-biopharm). Extraction solvent consisted in 125 mL of MeOH/H_2_O (90:10) and 2.5 g of NaCl. An aliquot of 5 mL 10-fold diluted in PBS (1.37 mol L^−1^) was passed through the column. Precolumn derivatization was performed after the evaporation step using 50 µL of 4-dimethylaminopyridine (107700, Sigma-Aldrich, St. Louis, Mo, USA) and 50 µL of 1-anthroyl cyanide (017-12101, FUJIFILM (Wako Pure Chemical Corporation, Osaka, Japan) both at 1 mg mL^−1^ in toluene (TX0737, Sigma-Aldrich). Gradient mode started at 70:30 CH_3_CN, Solvent A/H_2_O, Solvent B as per chromatographic conditions. The rest of the program was as follows: at 5 min 70% A, at 15 min 70% A, at 25 min 85% A, at 27 min 100% A, at 32 min 100% A, and at 35 min 70% A. Flow rate was set at 1 mL min^−1^. Adduct fluorescence was measured at λ_ex_ = 381 and λ_em_ = 470 nm. Linear calibration curves ranging from 125.00 to 1000.00 µg L^−1^ were prepared during quantification. The limit of quantification for T-2 and HT-2, was 5.00 and 3.00 μg kg^−1^, respectively. 

#### 5.4.3. ZEA

Extraction was performed using 100 mL of CH_3_CN/H_2_O 60:40 and an EASI-EXTRACT^®^ ZEARALENONE IAC (R-biopharm). Isocratic mode using a 40:10:50 CH_3_CN/CH_3_OH/H_2_O mixture at a flow rate of 0.7 mL min^−1^ was used as per chromatographic conditions. ZEA natural fluorescence (at λ_ex_ = 236, λ_em_ = 464 nm) was exploited for detection purposes. Linear calibration curves ranging from 300.00 to 1200.00 µg L^−1^ were prepared during quantification. The limit of quantification was 0.072 μg kg^−1^.

#### 5.4.4. FB_1_ and FB_2_

Extraction was performed using 100 mL of CH_3_CN/MeOH/H_2_O (25:25:50) and FUMONIPREP^®^ IAC (R-biopharm). Fumonisin derivatization was based on the reaction with *o*-phthalaldehyde (Millipore Sigma, P0657) and 2-mercaptoethanol (Millipore Sigma, 97622) as stated on the reference method. However, pre-column derivatization was performed in situ in the autosampler injector, according to Bartolomeo and Maisano (2006), but increasing the sample and OPA reagent volume 5-fold. Adduct fluorescence was measured at λ_ex_ = 335 and λ_em_ = 440 nm. Isocratic mode using MeOH/0.1 mol L^−1^ NaH_2_PO_4_ (77:23), adjusted to apparent pH 3.3 with H_3_PO_4_, was used at a 0.8 mL min^−1^ flow rate. The limit of quantification was 0.05 μg kg^−1^ for both FB_1_ and FB_2_.

#### 5.4.5. OTA

Extraction was performed using 100 mL of CH_3_CN/H_2_O 60:40 and an OCRAPREP^®^ IAC column. OTA elution from column and resuspension after evaporation was achieved using a 98:2 MeOH and acetic acid solution to ensure OTA protonation. Isocratic mode using a 50:50 H_2_O/CH_3_CN mixture using 0.2 mol L^−1^ trifluoroacetic acid, pH = 2.1 (74564 Millipore Sigma) at a flow rate of 0.7 mL min^−1^ was used as per chromatographic conditions. OTA natural fluorescence (at λ_ex_ = 247, λ_em_ = 480 nm) was exploited for detection purposes. Linear calibration curves ranging from 2.50 to 40 µg L^−1^ were prepared during quantification. The limit of quantification was 0.011 μg kg^−1^.

#### 5.4.6. AFM_1_ in Milk and Butter

AflaStar^®^ M_1_ (Romer Labs Diagnostic GmbH, Tulln an der Donau, Austria) columns were used for sample extraction. An exact 50 mL of raw or processed milk, previously homogenized and filtered by gravity over an ashless filter paper, was transferred to the IAC column. After a washing step using 3× 10 mL of water, the columns were left to dry and eluted using MeOH and concentrated as described above in 5.4.1. Isocratic mode using a 10:35:55 CH_3_CN/CH_3_OH/H_2_O mixture at a flow rate of 0.6 mL min^−1^ was used as per chromatographic conditions. AFM_1_ natural fluorescence (at λ_ex_ = 365, λ_em_ = 455 nm) was exploited for detection purposes. Linear calibration curves ranging from 0.50 to 2.00 µg L^−1^ were prepared during quantification. The limit of quantification was 0.014 μg kg^−1^.

In the case of the butter samples, the preparation was performed according to the method in [84]. Briefly, 25 mL of aqueous methanol (70 mL/100 mL) was added to 5 g of butter. Afterwards, the solution was extracted by mixing gently for 10 min at room temperature using sonication. The extract was filtered through a paper filter, and 15 mL of distilled water was added to 5 mL of filtered solution. After that, 0.25 mL of Tween 20 were added and dispersed for 2 min, followed by the entire amount of the sample solution (20 mL) passing over the IAC.

### 5.5. Data Analysis

For Table 1, Table 2 and Table 3, prevalence is expressed as the ratio between the total of assays above the limit of detection and the total of assays performed for each toxin. Descriptive statistics displayed in Table 1 are expressed without considering samples below the limit of detection. Heat maps used in Figure 1 were rendered using ArcGIS Pro v2.2 (Esri^TM^, Redlands, CA, USA). For each contaminant, Spearman Rank Order tests were applied to assess the association among the toxin concentration and climatic variables (i.e., precipitation, rainy days and temperature). In this particular case, toxin levels below the limit of detection were considered zero for association purposes; this analysis was performed using SigmaPlot 14 (Systat Software Inc., San Jose, CA, USA). Sampling date was linked to mean monthly values and data were retrieved from the closest climatological station to the sampling region. Meteorological data were provided by the Costa Rican National Weather Service (https://www.imn.ac.cr/boletin-meteorologico). 

## Figures and Tables

**Table 1:** Mycotoxin presence and concentration in animal feedstuff commercialized in Costa Rica.

Year	Sample Numbers, *n*						Prevalence (%) (Samples over the Limit of Detection)	Average ± Standard Deviation ^b^	Median ^b^
		Concentration Range, µg kg^−1^ ^a^					Concentration, µg kg^−1^		
		*x* < LoD	*x* < 250	250 ≤ *x* < 500	500 ≤ *x* < 1000	*x* ≥ 1000			
*Zearalenone*									
2013	47	19	27	1	0	0	59.6	30 ± 80	10
2014	57	8	49	0	0	0	86.0	15 ± 15	11
2015	62	44	18	0	0	0	29.0	33 ± 62	7
2016	99	79	12	6	2	0	20.0	180 ± 225	44
2017	61	35	8	9	3	6	42.6	1055 ± 1587	392
Total	335	194	114	16	5	6	42.1	236 ± 784	18
*3-acetyldeoxynivalenol*									
2015	67	53	7	3	2	2	20.9	1602± 4238	251
2016	91	74	7	0	3	7	18.7	1691 ± 2757	594
2017	54	40	7	3	3	1	25.9	400 ± 398	275
*Deoxynivalenol*									
Total	212	167	21	6	8	10	21.2	1261 ± 2909	295
2013	40	11	0	7	10	12	72.5	10,439 ± 29,521	830
2014	87	18	15	32	10	12	79.3	966 ± 2442	372
2015	81	44	9	11	10	7	45.7	703 ± 916	467
2016	94	54	13	7	7	11	42.5	1150 ± 1888	355
2017	61	24	10	13	8	6	60.7	4147 ± 18,710	400
Total	363	151	47	70	45	48	58.4	2822 ± 13,805	439
		*x* < LoD	5 < *x*	10 ≤ *x* < 25	25 ≤ *x* < 50	*x* ≥ 50			
*Ochratoxin A*									
2013	49	41	8	0	0	0	16.3	2 ± 2	2
2014	101	59	42	0	0	0	41.6	1 ± 2	1
2015	64	15	49	0	0	0	76.7	11 ± 23	1
2016	95	68	26	0	0	1	28.4	90 ± 346	3
2017	50	30	20	0	0	0	40.0	32 ± 63	5
Total	360	214	145	0	0	0	40.6	25 ± 152	1
*T-2 toxin*									
2013	48	23	12	8	1	4	52.1	406 ± 467	273
2014	126	49	56	15	6	0	61.1	171 ± 227	61
2015	91	66	24	0	1	0	27.5	39 ± 130	9
2016	93	77	15	0	0	1	17.2	180 ± 509	15
2017	47	36	11	0	0	0	23.4	20 ± 18	13
Total	406	251	119	23	8	5	47.0	177 ± 317	47
*HT-2 toxin*									
2014	47	17	16	6	4	4	63.8	1113 ± 2661	217
2015	86	66	14	3	2	1	23.2	257 ± 399	151
2016	92	56	29	3	1	3	39.1	199 ± 359	53
2017	44	33	10	1	0	0	25.0	108 ± 71	103
Total	269	172	66	13	7	8	36.1	463 ± 1495	115
		x < LoD	x < 1250	1250 ≤ x < 2500	2500 ≤ x < 5000	x ≥ 5000			
*Fumonisin B_1_*									
2013	31	29	1	0	1	0	6.4	1691 ± 2117	1670
2014	35	27	3	1	2	2	22.9	3814 ± 3793	3625
2015	24	10	6	2	1	5	58.3	4551 ± 5774	3865
2016	88	54	13	5	4	12	38.6	3468.48 ± 7159	740
2017	59	43	11	2	3	0	27.1	203.64 ± 48	230
Total	237	163	34	10	11	19	31.2	3390 ± 5505	3110
*Fumonisin B_2_*									
2014	8	0	4	0	0	4	100.0	2794 ± 2252	2830
2015	11	1	3	2	2	3	90.9	6635 ± 9404	2010
2016	33	21	10	0	0	2	36.4	9931 ± 18,380	1793
2017	29	24	5	0	0	0	17.2	866 ± 1131	175
Total	81	46	22	2	2	9	43.2	6353 ± 13,559	1560

^a^ Ranges based on guidance values for mycotoxins in animal feeds within the European Union (Commission Recommendations 2006/576/EC and 2013/165/EU). [37,38]. ^b^ Values are calculated based on the number of samples above limit of detection.

**Table 2:** Mycotoxin contamination levels for feed ingredients. ^a^

Average ± Standard Deviation	Median	Sample Numbers above Guidance Value, n	Prevalence, % (Sample Totals Analyzed by Toxin) ^c^
Concentration, µg kg^−1^			
**Corn and Byproducts**			
*Deoxynivalenol (12,000 µg kg^−1^)* ^b^			
650 ± 346	440	0	61.1 (36)
*Fumonisin B_1_ (60,000 µg kg^−1^ sum FB_1_/FB_2_)* ^b^			
18,280 ± 16,016	3230	0	35.9 (39)
*HT-2 toxin (500 µg kg^−1^ sum T-2/HT-2)* ^b^			
493 ± 927	84	3	62.6 (24)
*Ochratoxin A (250 µg kg^−1^)* ^b^			
18 ± 45	1	0	25.6 (39)
*T-2 toxin*			
195 ± 256	53	3	55.7 (61)
*Zearalenone (3000 µg kg^−1^)* ^b^			
314 ± 895	15	2	71.1 (45)
**Soybean Meal (there is no recommended Guidelines) ^b^**			
*Deoxynivalenol*			
188 ± 69	200	Not applicable	60.0 (5)
*Fumonisin B_1_*			
3045 ± 1096	3045	Not applicable	100.0 (2)
*HT-2 toxin*			
5013 ± 6542	2140	Not applicable	50.0 (6)
*T-2 toxin*			
120 ± 141	50	Not applicable	61.5 (13)
**Wheat and Byproducts**			
*Deoxynivalenol (8000 µg kg^−1^)* ^b^			
20,290 ± 52,867	890	1	100.0 (8)
*Fumonisin B_1_ (60,000 µg kg^−1^ sum FB_1_/FB_2_)* ^b^			
2050 ± 2234	576	0	50.0 (4)
*HT-2 toxin (500 µg kg^−1^ sum T-2/HT-2)* ^b^			
44 ± 50	65	0	66.7 (3)
*Ochratoxin A (250 µg kg^−1^)* ^b^			
2 ± 2	1	0	50.0 (4)
*T-2 toxin*			
64 ± 61	54	0	75.0 (8)
*Zearalenone (2000 µg kg^−1^)* ^b^			
12 ± 14	5	0	28.6 (7)
**Rice and Byproducts**			
*3-acetyldeoxynivalenol (there is no recommended guideline)* ^c^			
351 ± 79	351	Not applicable	50.0 (4)
*Deoxynivalenol (8000 µg kg^−1^)* ^b^			
890 ± 400	1101	0	60.0 (5)
**Palm Oil and Byproducts (there is no recommended guidelines) ^b^**			
*Deoxynivalenol*			
400 ± 359	286	Not applicable	55.6 (18)
*T-2 toxin*			
330 ± 625	58	Not applicable	61.5 (13)
*Zearalenone*			
19 ± 18	13	Not applicable	30.0 (10)
**Fruit Pulps and Peels (there is no recommended guidelines) ^b^**			
*3-acetyldeoxynivalenol*			
2204 ± 2394	2104	Not applicable	40.0 (10)
*Deoxynivalenol*			
21,249 ± 41,315	2160	Not applicable	50.0 (14)
*Fumonisin B_1_*			
16,564 ± 18,916	7010	Not applicable	34.7 (32)
*Fumonisin B_2_*			
10,100 ± 13,096	16,564	Not applicable	50.0 (4)
*Ochratoxin A*			
4 ± 7	1	Not applicable	50.0 (12)
*T-2 toxin*			
330 ± 464	50	Not applicable	13.3 (15)
*Zearalenone*			
43 ± 31	21	Not applicable	11.8 (17)
**Forages, Silages, and Hay (there is no recommended guidelines) ^b^**			
*3-acetyldeoxynivalenol*			
476 ± 431	335	Not applicable	54.5 (22)
*Deoxynivalenol*			
655 ± 514	410	Not applicable	66.7 (30)
*Fumonisin B_1_*			
11,883 ± 6917	7740	Not applicable	9.4 (32)
*Fumonisin B_2_*			
3985 ± 5310	1020	Not applicable	22.2 (9)
*HT-2 toxin*			
124 ± 132	126	Not applicable	25.0 (16)
*Ochratoxin A*			
15 ± 30	2	Not applicable	54.5 (22)
*T-2 toxin*			
119 ± 177	25	Not applicable	30.4 (23)
*Zearalenone*			
314 ± 724	27	Not applicable	37.5 (24)
**Others (there is no recommended guidelines) ^b^**			
*Deoxynivalenol*			
610 ± 519	567	Not applicable	38.5 (13)
*Fumonisin B_1_*			
4931 ± 5994	693	Not applicable	66.7 (3)
*HT-2 toxin*			
193 ± 136	197	Not applicable	75.0 (12)
*Ochratoxin A*			
1 ± 3	1	Not applicable	56.3 (64)
*T-2 toxin*			
6 ± 3	6	Not applicable	38.5 (13)
*Zearalenone*			
9 ± 5	9	Not applicable	18.2 (11)

^a^ Toxins detected only once for a specific matrix type were not included. ^b^ Data in parentheses indicate the permitted maximum or recommended toxin concentrations according to EU Commission Recommendations (2006/576/EC) [37] and (2013/165/EU) [34]. ^c^ Prevalence is calculated based on the number of samples above limit of detection.

**Table 3:** Mycotoxin contamination levels for compound animal feed. ^a^

Average ± Standard Deviation	Median	Sample Numbers above Recommended Guidance Value, n	Prevalence, % (Sample Totals Analyzed by Toxin) ^d^
Concentration, µg kg^−1^			
**Beef Cattle Feed**			
*3-acetyldeoxynivalenol (there is no recommended guideline)* ^c^			
166 ± 159	77	Not applicable	42.9 (7)
*Deoxinivalenol (5000 µg kg^−1^)* ^c^			
988 ± 1371	530	0	70.0 (10)
*Fumonisin B_1_ (50,000 µg kg^−1^ sum FB_1_/FB_2_)* ^c^			
8912 ± 13,416	3305	0	88.9 (9)
*Fumonisin B_2_*			
4020 ± 4921	134	0	66.7 (3)
*HT-2 toxin (250 µg kg^−1^ sum T-2/HT-2* ^c^			
442 ± 736	20	1	37.5 (8)
*T-2 toxin*			
128 ± 126	110	1	30.0 (10)
*Ochratoxin A (there is no recommended guideline)* ^c^			
19 ± 22	12	Not applicable	44.4 (9)
*Zearalenone (500 µg kg^−1^)* ^c^			
269 ± 216	157	0	57.1 (7)
Ingredients ^b†^: cornmeal (no restriction), soybean meal (no restriction), DDGG (12–15 g/100 g), palm kernel meal (max 10–15 g/100 g), wheat middlings (max 10–20 g/100 g), rice bran and polishings (max 10–20 g/100 g), soybean hulls (max 10 g/100 g), citrus pulp (10 g/100 g).			
**Dairy cattle Feed (Adults and Heifers)**			
*3-acetyldeoxynivalenol (there is no recommended guideline)* ^c^			
1843 ± 4135	218	Not applicable	19.0 (105)
*Deoxynivalenol (5000 µg kg^−1^)* ^c^			
1578 ± 4613	338	5	55.1 (147)
*Fumonisin B_1_ (50,000 µg kg^−1^ sum FB_1_/FB_2_* ^c^			
6171 ± 7908	1480	0	44.4 (144)
*Fumonisin B_2_*			
3838 ± 5913	2310	0	43.2 (44)
*HT-2 toxin (250 µg kg^−1^ sum T-2/HT-2* ^c^			
207 ± 282	106	13	35.6 (132)
*Ochratoxin A (there is no recommended guideline* ^c^			
55 ± 259	1	Not applicable	35.0 (140)
*T-2 toxin*			
184 ± 351	40	10	27.5 (171)
*Zearalenone (500 µg kg^−1^* ^c^			
215 ± 810	16	6	44.0 (150)
Ingredients ^b†^: cornmeal (no restriction), soybean meal (no restriction), DDGG (12–15 g/100 g), palm kernel meal (max 10–15 g/100 g), wheat middlings (max 10–20 g/100 g), rice bran and polishings (max 10–20 g/100 g), soybean hulls (max 10 g/100 g), citrus pulp (10 g/100 g).			
**Poultry Feed**			
*Deoxynivalenol (5000 µg kg^−1^)* ^c^			
1550 ± 2327	405	2	71.4 (14)
*Fumonisin B_1_ (20,000 µg kg^−1^ sum FB_1_/FB_2_)* ^c^			
17,147 ± 33,569	3860	1	70.0 (10)
*Fumonisin B_2_*			
436 ± 467	835	0	80.0 (5)
*HT-2 toxin (250 µg kg^−1^ sum T-2/HT-2)* ^c^			
353 ± 284	208	1	33.3 (15)
*Ochratoxin A (100 µg kg^−1^)* ^c^			
31 ± 48	11	1	44.4 (9)
*T-2 toxin*			
316 ± 462	67	5	51.7 (29)
*Zearalenone (there is no recommended guideline)* ^c^			
75 ± 117	28	Not applicable	50.0 (10)
Ingredients^b†^: corn meal (no restriction), soybean meal (no restriction), DDGG (max 10–15 g/100 g), palm kernel meal (3–3.5 g/100 g), wheat middlings (max 3–3.5 g/100 g), rice bran and polishings (max 3–3.5 g/100 g), soybean hulls (max 3–3.5 g/100 g).			
**Pet Food (Cat and Dog Dry Food)**			
*Deoxynivalenol (2000 µg kg^−1^)* ^c^			
940 ± 1317	470	0	50.0 (14)
*Fumonisin B1 (5000 µg kg^−1^ sum FB1/FB2)* ^c^			
143,560 ± 479,783	3570	6	93.3 (15)
Ingredients ^b†^: cornmeal (max 50 g/100 g), DDGG (max 25 g/100 g), palm kernel meal, wheat middlings (max 20 g/100 g), rice meal and bran (max 20 g/100 g).			
**Swine Feed (Lactating and Gestating Sows and Pig Grower)**			
*Deoxynivalenol (900 µg kg^−1^)* ^c^			
6302 ± 14,932	590	6	76.5 (17)
*Fumonisin B_1_ (5000 µg kg^−1^ sum FB_1_/FB_2_)* ^c^			
20,042 ± 35,978	3124	2	55.6 (9)
*Fumonisin B_2_*			
376 ± 472	376	0	40.0 (5)
*HT-2 toxin (250 µg kg^−1^ sum T-2/HT-2)* ^c^			
3409 ± 4738	3409	1	28.6 (7)
*T-2 toxin*			
183 ± 187	88	3	46.7 (15)
*Zearalenone (100 µg kg^−1^)* ^c^			
518 ± 1327	37	2	44.4 (18)
Ingredients ^b†^: cornmeal (no restriction), soybean meal (no restriction), DDGG (max 10 g/100 g), palm kernel meal (max 10 g/100 g), wheat middlings (max 20–25 g/100 g), rice bran and polishing (max 20–25 g/100 g), soybean hulls (no restriction).			
**Fish Feed**			
*Deoxynivalenol (500 µg kg^−1^)* ^c^			
570 ± 318	635	2	25.0 (16)
*Fumonisin B_1_ (10,000 µg kg^−1^ sum FB_1_/FB_2_)* ^c^			
10,851 ± 10,781	1565	2	52.4 (21)
*Ochratoxin A (there is no recommended guideline)* ^c^			
3 ± 5	1	Not applicable	66.7 (24)
	*T-2 toxin*		
4 ± 4	3	0	35.0 (20)
*Zearalenone (there is no recommended guideline)* ^c^			
84 ± 122	35	Not applicable	25.0 (16)
Ingredients^b†^: cornmeal (max 15 g/100 g), soybean meal (max 75 g/100 g), DDGG, palm kernel meal (max 30 g/100 g), wheat middlings (max 20 g/100 g), rice meal and bran (max 15 g/100 g), soybean hulls.			
**Horse Feed**			
*Deoxynivalenol (5000 µg kg^−1^)* ^c^			
740 ± 295	580	0	50.0 (6)
*Fumonisin B_2_*			
3355 ± 2623	3355	1	66.7 (3)
*HT-2 toxin (250 µg kg^−1^ sum T-2/HT-2)* ^c^			
52 ± 26	52	0	40.0 (5)
*Ochratoxin A (there is no recommended guideline)* ^c^			
95.36 ± 47.43	95	Not applicable	33.3 (6)
*T-2 toxin*			
49 ± 60	49	0	33.3 (6)
Ingredientsb†: cornmeal (max 45 g/100 g), soybean meal (max 13 g/100 g), DDGG (max 20 g/100 g), palm kernel meal, wheat middlings (max 25 g/100 g), rice bran, soybean hulls (max 20 g/100 g).			

^a^ Toxins detected only once for a specific matrix type were not included. ^b^ Plant-derived constituents according to guaranteed labels. Data in parentheses indicate maximum inclusion recommended for each ingredient during feed formulation. ^†^ Data compiled from [15,39,40,41,42]. ^c^ Data in parentheses indicate maximum permitted or recommended toxin concentrations according to EU Commission Recommendations (2006/576/EC) [37] and (2013/165/EU) [38]. ^d^ Prevalence is calculated considering the number of samples above limit of detection.

**Table 4:** Seasonal prevalence and behavior per toxin.

Concentration, mg kg^−1^			
Season ^a^	Positive Samples, *n* (Prevalence, %)	Average ± SD	Maximum
3-ADON			
Rainy Season	36/145 (24.8)	2 ± 3	16
DON			
Dry Season	57/101 (56.4)	3 ± 7	52
Rainy Season	130/229 (56.8)	17 ± 161	1830
FB_1_			
Dry Season	29/97 (29.9)	7 ± 12	40
Rainy Season	111/226 (49.1)	7 ± 13	77
FB_2_			
Dry Season	9/21 (42.9)	4 ± 8	23
Rainy Season	25/56 (44.6)	3 ± 4	19
HT-2 toxin			
Rainy Season	96/180 (53.3)	1 ± 2	11
T-2 toxin			
Dry Season	54/145 (37.2)	< 1	2
Rainy Season	94/248 (37.9)	< 1	1
OTA, μg kg^−1^			
Dry Season	31/112 (27.7)	7 ± 24	137
Rainy Season	88/204 (43.1)	37 ± 193	1810
ZEA			
Dry Season	46/94 (48.9)	1 ± 1	6
Rainy Season	90/228 (39.5)	1 ± 6	4
Overall Months with Higher Levels and Prevalence			
3-ADON	April and May	DON	No clear distribution
FB_1_	June, July, and September	FB_2_	April, June, and September
HT-2 toxin	October and November	T-2 toxin	No clear distribution
OTA	May and September	ZEA	May, July, and October

^a^ Dry season and rainy season defined as per mean precipitation, the former exemplified by the months between December and April where *x* < 80 mm rain.

**Table 5:** Mycotoxin co-occurrence in the sample totals.

Number of Toxins Simultaneously Present	2	3		4		5		6	7
**Samples, *n* (Incidence, %)**	141/279 ^a^ (50.54)	81/279 (29.0)		36/279 (12.9)		17/279 (6.1)		1/279 (0.4)	3/279 (1.1)
**Toxin/Metabolite**	Sample Numbers with the toxin present, *n*		Co-occurrence, *n*				Incidence, %		
DON/3-ADON	177		18				10.2		
FB_1_/FB_2_	137		23				16.8		
T-2/HT-2 toxin	155		66				42.6		
Toxin Co-occurrence with OTA	**Sample Numbers, *n***				**Incidence, %**				
DON + HT-2 toxin + ZEA	1				1.0				
DON + 3-ADON + FB_1_ + ZEA	1				1.0				
T-2 toxin + FB_1_ + ZEA	1				1.0				
DON + FB1 + FB2 + ZEA	1				1.0				
3-ADON	1				1.0				
DON + 3-ADON + T-2 toxin + FB_1_	1				1.0				
DON + HT-2 toxin + FB_1_ + ZEA	2				2.0				
T-2 toxin + HT-2 toxin + FB_1_ + ZEA	2				2.0				
DON + 3-ADON + T-2 toxin + HT-2 T-2 toxin + FB_1 +_ FB_2_ + ZEA	2				2.0				
FB1 + ZEA	2				2.0				
T-2/HT-2 toxin + ZEA	3				2.9				
T-2 toxin + FB_1_	3				2.9				
DON + T-2 toxin + HT-2 toxin	4				3.9				
DON + ZEA	4				3.9				
DON + T-2 + FB_1_ + ZEA	6				5.9				
DON	7				6.9				
HT-2 toxin	8				7.8				
T-2 toxin	10				9.8				
FB_1_/FB_2_	12				11.8				
DON + FB_1_	14				13.7				
ZEA	17				16.7				

^a^ Corresponds to the total number of samples in which ≥ 2 simultaneous toxins occurred.

**Table 6:** Prevalence and epidemiological data regarding AFM_1_ in fresh bovine milk for four years.

Concentration ^b^, ng mL^−1^							
Year	Positive Samples, *n* (Prevalence, %) ^a^	Samples > 0.05 µg kg−^1^, n (%)	Samples > 0.5 µg kg^−1^, *n* (%)	Average ± SD	Median	Maximum	Minimum
2017	24/38 (63.2)	16 (42.1)	0	0.083 ± 0.076	0.061	0.334	0.013
2016	8/27 (29.6)	2 (7.4)	0	0.042 ± 0.030	0.032	0.109	0.014
2015	34/73 (46.6)	16 (21.9)	3 (4.1)	0.154 ± 0.236	0.057	0.989	0.017
2014	32/45 (71.1)	11 (24.4)	0	0.042 ± 0.038	0.030	0.164	0.005
Overall	98/183 (53.5)	45 (45.9)	3 (3.1)	0.091 ± 0.155	0.049	0.989	0.005
Dry season ^c^	28/45 (62.2)	14 (50.0)	0	0.075 ± 0.105	0.050	0.485	0.005
Rainy season ^c^	69/138 (50.0)	34 (49.3)	3 (4.3)	0.098 ± 0.172	0.049	0.989	0.005
Overall months with higher levels and prevalence				March, August, and September			

^a^ Prevalence understood as the number of samples > Limit of quantificaction of 0.014 µg kg^−1^. ^b^ Values obtained using only positive samples, i.e., > limit of detection. ^c^ Dry season and rainy season defined as per mean precipitation, the former defined by the months between December and April where *x* < 80 mm rain.

## Appendix A

**Table A1:** Indicative Levels for T-2 and HT-2 in Cereals and Cereal products according to UE ^a^.

Matrix	Indicative Levels for the Sum of T-2 and HT-2 (µg kg^−1^) from Which Onwards/above Which Investigations Should be Performed, Certainly in Case of Repetitive Findings
Unprocessed Cereals	
Barley (including malting barley) and maize	200
Oats (with husk)	1000
Wheat, rye and other cereals	100
Cereal Products for Feed and Compound Feed	
Oat milling products (husks)	2000
Other cereal products	500
Compound feed with the exception of feed for cats	250

^a^ Based on Reference [38] and according to 2013/165/EU. Please see notes contained in each recommendation.

**Table A2:** Relevant guidance values for each mycotoxin in products intended for animal feed according to UE ^a^.

Mycotoxin	Products Intended for Animal Feed	Guidance Value in mg kg^−1^ Relative to a Feedstuff with a Moisture Content of 12 g/100 g
Deoxynivalenol	Feed materials	
	Cereals and cereal products with the exception	8
	Cereals and cereal products with the exception	12
	Compound feed (exception of compound feed for pigs, calves (<4 months), lambs, kids and dogs)	5
	Compound feed for pigs	0.9
	Compound feed for calves (<4 months), lambs, kids and dogs	2
Zearalenone	Feed materials	
	Cereals and cereal products with the exception of maize byproducts	2
	Maize byproducts	3
	Compound feed for:	
	Piglets, gilts (young sows), puppies, kittens, dogs and cats for reproduction	0.1
	Adult dogs and cats other than for reproduction	0.2
	Sows and fattening pigs	0.25
	Calves, dairy cattle, sheep (including lamb) and goats (kids)	0.5
Ochratoxin A	Feed materials	
	Cereals and cereal products	0.25
	Compound feed for	
	Pigs	0.05
	Poultry	0.1
	Cats and dogs	0.01
Fumonisin FB_1_ + FB_2_	Feed materials	
	Maize and maize products	60
	Compound feed for	
	Pigs, horses (*Equidae*), rabbits and pet animals	5
	Fish	
	Poultry, calves (<4 months), lambs and kids	20
	Adult ruminants (> 4 months) and mink	50
T2 + HT-2	Compound Feed for Cats	0.05

^a^ Based on Reference [37] and according to 2006/576/EC, 2016/1319, and definitions stated in 68/2013/EC. Please see notes contained in each recommendation.
